# Targeting nail psoriasis: IL-17A inhibitors demonstrate site-specific superiority over IL-23 inhibitor in a 24-week dermoscopy-guided real-world cohort

**DOI:** 10.3389/fimmu.2025.1573715

**Published:** 2025-04-08

**Authors:** Xiamei Yan, Minglan Shi, Bin Wang, Lihua Zeng, Huiwei Wang, Jialiang Shi, Yaqian Cui, Suchun Hou

**Affiliations:** ^1^ Department of Dermatology, Nanshan Maternity and Child Health Care Hospital, Shenzhen, China; ^2^ Department of Dermatology, Shenzhen Hospital, The University of Hong Kong, Shenzhen, China; ^3^ Department of Dermatology, Shenzhen University General Hospital, Shenzhen, China; ^4^ Department of Dermatology, Shenzhen Longhua People’s Hospital, Shenzhen, China

**Keywords:** nail psoriasis, dermoscopy, IL-17A inhibitors, IL-23 inhibitor, real-world cohort, NAPSI

## Abstract

**Objective:**

To compare the real-world clinical efficacy and safety of interleukin (IL)-17A inhibitors (secukinumab [SEC] and ixekizumab [IXE]) versus the IL-23 inhibitor guselkumab (GUS) in patients with nail psoriasis, with a focus on site-specific biologic therapeutic responses (nail matrix vs. nail bed) in a 24-week prospective observational cohort.

**Methods:**

This cohort enrolled 65 adult patients with plaque psoriasis and dermoscopy-confirmed nail involvement, stratified into three treatment groups: SEC (n=25), IXE (n=20), and GUS (n=20). Outcome assessments at baseline and week 24 included: Nail Psoriasis Severity Index (NAPSI) with domain-specific scoring (matrix/bed) by dermoscopic evaluation using a 10× polarized handheld device; Psoriasis Area and Severity Index (PASI), Body Surface Area (BSA); Dermatology Life Quality Index (DLQI). Safety was monitored through treatment-emergent adverse events (TEAEs).

**Results:**

(1) By week 24, PASI, BSA, DLQI and NAPSI scores had significantly decreased from baseline in all groups (*P*<0.001). (2) By week 24: SEC, IXE, and GUS groups saw nail matrix NAPSI score improvements of 65.9%, 60.5%, and 51.5%, with 68%, 55%, and 30% achieving NAPSI 60; Nail bed NAPSI score improvements were 58.8%, 68.6%, and 65.8%, with 28%, 65%, and 40% achieving NAPSI 60; Total NAPSI score improvements were 62.7%, 64.6%, and 53.7%, with 44%, 70%, and 30% achieving NAPSI 60. (3) All patients in the SEC and IXE groups achieved PASI 75, compared to 85% in the GUS group. SEC showed PASI 90 and PASI 100 response rates of 80% and 36%, while IXE of 60% and 30%. (4) TEAEs were mild, including: injection site reactions: 15% (IXE group); eczematous rashes: 8% (SEC group). No TEAEs were reported in the GUS group, and no serious adverse events occurred in any group.

**Conclusion:**

IL-17A inhibitors and the IL-23 inhibitor demonstrated significant efficacy in improving both nail and skin lesions in psoriasis. Notably, IL-17A inhibitors exhibited superior overall efficacy compared to IL-23 inhibitor. Specifically, SEC excelled in improving dermoscopic nail matrix changes, whereas IXE was more potent for nail bed pathology. All groups significantly improved patients’ life quality and exhibited good safety profiles.

## 1 Introduction

Psoriasis is an immune-mediated, chronic, recurrent, inflammatory, systemic disease triggered by the interplay of genetic and environmental factors. The disease affects up to 3% of the global population (1). It can affect not only the skin but also various other parts of the body, including the nails and joints. Psoriasis is notoriously challenging to be managed, frequently progressing into a lifelong condition (2), as its precise pathogenesis remains unclear and may involve genetic, infectious, immune, and psychosomatic factors.

Nail involvement is common among psoriasis patients, with studies indicating that 10%-82% experience nail lesions (3). Furthermore, the lifetime prevalence of nail involvement can reach as high as 80-90%, and notably, 5-10% of patients exhibit nail psoriasis (NP) without any accompanying skin involvement (3, 4). NP is strongly associated with psoriatic arthritis (PsA) and is considered a key component of PsA diagnostic criteria (5). It is estimated that 80%-90% of PsA patients exhibit nail involvement (6). Nail involvement is regarded as a sign of uncontrolled inflammation and a predictor of more severe psoriasis and/or joint involvement (1, 7). This may be linked to the distal interphalangeal (DIP) joint, a common site for PsA, where the extensor tendon attachment crosses the DIP joint, connecting to the nail root and matrix (7, 8). If left untreated, nail-related diseases can lead to irreversible joint damage (1). Additionally, nail lesions affect hand aesthetics and functionality, causing significant psychological distress, including anxiety and depression (9). Therefore, diagnosing, evaluating, and treating psoriatic nail disease is essential for improving patients’ overall well-being.

Nail psoriasis arises from the involvement of the nail matrix and/or nail bed. Matrix involvement can manifest as pitting, leukonychia, red spots in the lunula, and onychodystrophy, while bed involvement may present as oil-drop discoloration, onycholysis, subungual hyperkeratosis, and splinter hemorrhages (7, 10). NP with concurrent skin lesions is easy to diagnose, whereas isolated NP presents a diagnostic challenge. Current auxiliary diagnostic methods for nails, such as MRI, is limited by availability and high cost. Ultrasound heavily depends on the examiner’s skill and experience, and histopathological examination is invasive and often not well accepted by patients. Given the nail’s unique anatomical structure, dermatologists are continually seeking new diagnostic approaches. Dermoscopy, a non-invasive dermatological examination tool, provides valuable diagnostic and differential diagnostic evidence for various pigmented and non-pigmented skin diseases (11). With high-resolution imaging, dermoscopy allows for detailed observation of the nail plate, nail fold, nail matrix, nail bed, and vasculature.

The development and application of biologics, particularly monoclonal antibodies targeting specific inflammatory mediators such as tumor necrosis factor (TNF)-α, interleukin (IL)-17, IL-23, and IL-12/23, have fundamentally transformed the treatment landscape for moderate-to-severe psoriasis and its associated nail disease. Biologics have demonstrated significant improvements in skin symptoms and have proven effective for psoriatic nails in real-world clinical practice (12–19). However, individual responses to biologics vary among patients with nail psoriasis. The differential impacts of IL-17A and IL-23 inhibitors on nail matrix versus bed pathology remain underexplored in real-world settings. Is there a correlation between the types of nail psoriasis and the efficacy of biologics? What clinical indicators can help doctors choose a more appropriate treatment? Therefore, we initiated this study to research the correlation between different dermoscopic phenotypes of nail psoriasis and the efficacy of biologic treatments.

## 2 Materials and methods

### 2.1 Populations of study

This prospective cohort study was conducted at the Dermatology Department of the University of Hong Kong-Shenzhen Hospital between December 2022 and December 2023. A total of 65 adult patients with plaque psoriasis and dermoscopy-confirmed nail involvement were enrolled and randomly assigned to three groups: 25 patients in secukinumab (SEC) group; 20 patients in ixekizumab (IXE) group; 20 patients in guselkumab (GUS) group. Baseline characteristics were balanced across groups (P > 0.05), further details are provided in **Supplementary Table S1**.

### 2.2 Study protocol

The severity of nail psoriasis was assessed by a blinded dermatologist at baseline and week 24 using the Nail Psoriasis Severity Index (NAPSI) score, with nail bed and matrix abnormalities evaluated under a 10× handheld dermoscope (DermLite^®^ DL4). Concurrently, Psoriasis Area and Severity Index (PASI), Body Surface Area (BSA), and Dermatology Life Quality Index (DLQI) scores were recorded. Clinical efficacy was determined based on the NAPSI improvement rate, categorized as: (1) ineffective: <30% improvement in matrix, bed, or total NAPSI; (2) improved: 30%-59% improvement; (3) marked improvement: 60%-99% improvement; (4) cured: 100% improvement. The clinical efficacy rate was calculated as: (number of marked improvement cases + number of cured cases)/total number of cases × 100%. The biologic regimens of the three groups were as follows: SEC group: 25 patients receiving subcutaneous SEC 300 mg at weeks 0, 1, 2, 3, 4, followed by 300 mg monthly; IXE group: 20 patients received subcutaneous IXE administered as a 160 mg loading dose (two 80 mg injections) at week 0, followed by 80 mg injections at weeks 2, 4, 6, 8, 10, and 12, then transitioning to a maintenance dose of 80 mg every 4 weeks; GUS group: 20 patients treated with GUS 100 mg at weeks 0, 4, 12 and 20.

### 2.3 Statistical analysis

Statistical analyses were performed using SPSS 29.0 (IBM, USA), and data visualization was conducted with GraphPad Prism 9.5.0 (GraphPad Software, USA).

## 3 Results

### 3.1 Efficacy of psoriatic nail disease treatment

#### 3.1.1 Nail matrix involvement characteristics and NAPSI

**Figure 1:**
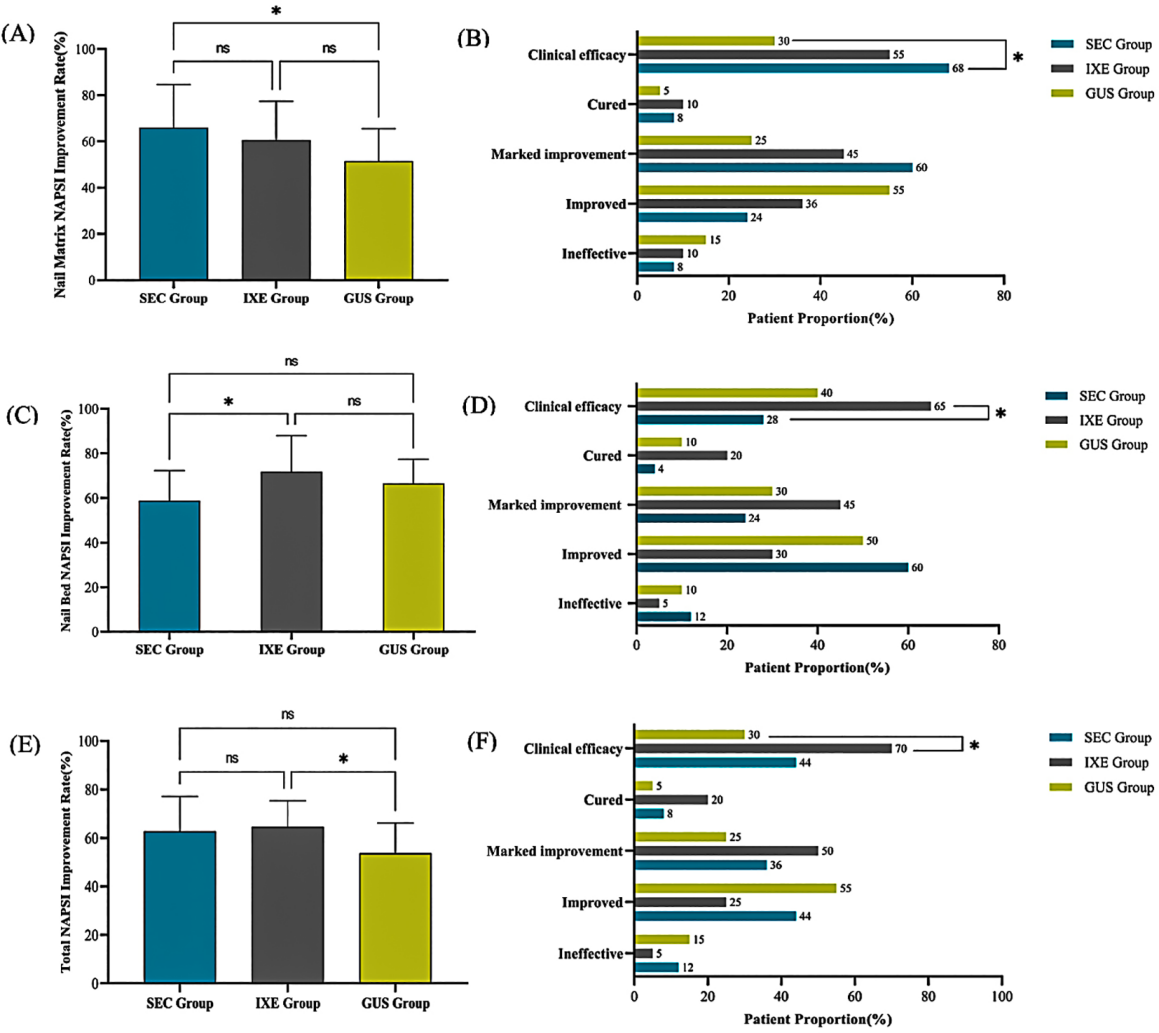
**(A)** Nail Matrix NAPSI Improvement Rate. **(B)** Nail Matrix NAPSI Improvement Levels. **(C)** Nail Bed NAPSI Improvement Rate. **(D)** Nail Bed NAPSI Improvement Levels. **(E)** Total NAPSI Improvement Rate. **(F)** Total NAPSI Improvement Levels. *P<0.05, ns, no statistical significance.

The nail matrix NAPSI scores and their subcomponent scores significantly decreased from baseline in all groups (*P* < 0.001; **Table 1**). At week 24, the mean improvement rates in nail matrix NAPSI scores were 65.9 ± 18.7% (SEC), 60.5 ± 16.9% (IXE), and 51.5 ± 14.0% (GUS), with significant between-group differences (*P* < 0.05). Pairwise comparisons revealed that SEC outperformed GUS (*P* = 0.017), while no significant differences were observed between IXE and GUS (*P* = 0.284) or SEC and IXE (*P* = 0.844) (**Figure 1A**).

**Table 1:** Nail matrix characteristics and scores.

Nail matrix information	SEC group (n=25)	IXE group (n=20)	GUS group (n=20)	*F/H/X^2^* value	*P* value
pitting, median (P25, P75)	19.0 (8.5,38.5)	22.0 (15.0,25.0)	28.0 (12.0,40.0)	0.847	0.655
pitting (week 24), median (P25, P75)	6.0 (1.0,17.0)	9.0 (2.0,13.0)	13.0 (4.0,27.0)	4.286	0.117
onychodystrophy, median (P25, P75)	14.0 (7.0,36.0)	15.0 (3.0,25.0)	11.0 (4.0,31.0)	0.143	0.931
onychodystrophy (week 24), median (P25, P75)	5.0 (2.0,12.5)	6.0 (1.0,11.0)	3.0 (1.0,10.0)	0.604	0.739
leukonychia, median (P25, P75)	9.5 (5.0,22.0)	5.0 (2.5,13.0)	6.5 (3.0,16.0)	0.976	0.614
leukonychia (week 24), median (P25, P75)	3.0 (0.0,7.0)	1.5 (0.0,8.0)	2.0 (0.0,6.0)	0.356	1.000
red spots in the lunula, median (P25, P75)	10.0 (2.5,18.5)	8.0 (1.0,16.0)	11.0 (4.0,17.0)	0.632	0.729
red spots in the lunula (week 24), median (P25, P75)	1.5 (0.0,6.0)	2.0 (0.0,7.0)	4.0 (2.0,10.0)	3.333	0.189
nail matrix NAPSI, median (P25, P75)	47.0 (15.5,74.0)	35.0 (22.5,43.5)	36.5 (17.3,59.8)	0.150	0.712
nail matrix NAPSI (week 24), median (P25, P75)	12.0 (4.0,27.0)	12.5 (10.0,18.8)	15.5 (6.0,29.2)	3.331	0.535
nail matrix improvement rate (%), mean ± SD	65.9 ± 18.7	60.5 ± 16.9	51.5 ± 14.0	4.157	0.020
Ineffective, n (%)	2 (8.0)	2 (10.0)	3 (15.0)		0.881
Improved, n (%)	6 (24.0)	7 (35.0)	11 (55.0)	4.631	0.099
Marked improvement, n (%)	15 (60.0)	9 (45.0)	5 (25.0)	5.510	0.064
Cured, n (%)	2 (8.0)	2 (10.0)	1 (5.0)		1.000
Clinical efficacy, n (%)	17 (68.0)	11 (55.0)	6 (30.0)	6.515	0.038

Although no significant between-group differences were observed in overall improvement magnitudes (*P* > 0.05), the clinical efficacy rates (NAPSI 60 achievement) for nail matrix lesions differed markedly: 68% (SEC), 55% (IXE), and 30% (GUS). Pairwise comparisons revealed that SEC demonstrated a significantly higher clinical efficacy rate compared to GUS (*P* = 0.011), while no significant differences were observed between IXE and GUS (*P* = 0.110) or SEC and IXE (*P* = 0.371) (**Figure 1B**).

#### 3.1.2 Nail bed involvement characteristics and NAPSI

The nail bed NAPSI scores and their subcomponent scores significantly decreased from baseline in all groups (*P* < 0.001; **Table 2**). At week 24, the mean improvement rates in nail bed NAPSI scores were 58.8 ± 13.8% (SEC), 68.6 ± 11.5% (IXE), and 65.8 ± 8.9% (GUS), with significant between-group differences (*P* < 0.05). Pairwise comparisons revealed that the IXE outperformed SEC (*P* = 0.022), while no significant differences were observed between IXE and GUS (*P* = 1.000) or SEC and GUS (*P* = 0.159) (**Figure 1C**).

**Table 2:** Nail bed characteristics and scores.

Nail bed information	SEC group (n=25)	IXE group (n=20)	GUS group (n=20)	*F/H/X^2^* value	*P* value
onycholysis, median (P25, P75)	17.0 (11.0,20.0)	18.0 (12.0,20.0)	19.0 (13.5,26.0)	1.046	0.593
onycholysis (week 24), median (P25, P75)	6.0 (3.0,16.5)	3.0 (2.0,7.0)	5.5 (3.5,11.0)	4.226	0.121
splinter hemorrhages, median (P25, P75)	15.0 (1.0,19.0)	11.0 (5.0,18.0)	13.0 (1.5,16.0)	1.456	0.483
splinter hemorrhages (week 24), median (P25, P75)	4.0 (1.0,6.5)	1.0 (0.0,6.0)	4.0 (0.5,6.5)	1.019	0.601
subungual hyperkeratosis, median (P25, P75)	12.0 (1.0,20.0)	14.0 (2.0,18.0)	14.0 (4.5,21.5)	1.398	0.497
subungual hyperkeratosis (week 24), median (P25, P75)	1.5 (0.5,7.0)	0.0 (0.0,3.0)	3.0 (0.5,8.0)	1.288	0.525
oil-drop discoloration, median (P25, P75)	13.0 (3.0,7.5)	11.0 (2.0,12.0)	9.5 (3.5,17.0)	3.153	0.207
oil-drop discoloration (week 24), median (P25, P75)	2.0 (0.0,3.5)	1.0 (0.0,2.0)	1.5 (0.5,4.0)	1.992	0.369
Nail bed NAPSI, mean ± SD	42.2 ± 16.4	49.7 ± 20.8	57.5 ± 16.2	1.084	0.346
nail bed NAPSI (week 24), mean ± SD	14.0 ± 6.5	7.0 ± 3.2	16.4 ± 6.9	3.914	0.141
nail bed improvement rate (%), mean ± SD	58.8 ± 13.8	68.6 ± 11.5	65.8 ± 8.9	4.204	0.019
ineffective, n (%)	3 (12.0)	1 (5.0)	2 (10.0)		0.869
improved, n (%)	15 (60.0)	6 (30.0)	10 (50.0)	4.070	0.131
marked improvement, n (%)	6 (24.0)	9 (45.0)	6 (30.0)	2.311	0.315
cured, n (%)	1 (4.0)	4 (20.0)	2 (10.0)		0.275
clinical efficacy, n (%)	7 (28.0)	13 (65.0)	8 (40.0)	6.315	0.043

Although no significant between-group differences were observed in overall improvement magnitudes (*P* > 0.05), the clinical efficacy rates (NAPSI 60 achievement) for nail bed lesions differed markedly: 28% (SEC), 85% (IXE), and 40% (GUS). Pairwise comparisons revealed that IXE demonstrated a significantly higher clinical efficacy rate compared to SEC (*P* = 0.013), while no significant differences were observed between IXE and GUS (*P* = 0.113) or SEC and GUS (*P* = 0.396) (**Figure 1D**).

#### 3.1.3 Total NAPSI

The NAPSI scores for nail lesions in all group significantly decreased from baseline (*P* < 0.001) (**Table 3**). At week 24, the mean improvement rates in NAPSI scores for nail lesions were 62.7 ± 14.4% (SEC), 64.6 ± 10.8% (IXE), and 53.7 ± 12.4% (GUS), with significant between-group differences (*P* < 0.05). Pairwise comparisons revealed that the IXE outperformed GUS group (*P* =0.027), while no significant differences were observed between IXE and SEC (*P* =1.000) or SEC and GUS (*P* =0.065) (**Figure 1E**).

**Table 3:** Total NAPSI scores.

Nail lesions information	SEC group (n=25)	IXE group (n=20)	GUS group (n=20)	*F/H/X^2^* value	*P* value
total NAPSI, mean ± SD	85.1 ± 27.9	84.3 ± 32.4	91.0 ± 42.2	0.130	0.878
total NAPSI (week 24), mean ± SD	35.5 ± 16.6	30.9 ± 12.5	42.3 ± 17.8	0.905	0.410
total NASPI improvement rate (%), mean ± SD)	62.7 ± 14.4	64.6 ± 10.8	53.7 ± 12.4	4.249	0.019
ineffective, n (%)	3 (12.0)	1 (5.0)	3 (15.0)		0.694
improved, n (%)	11 (44.0)	5 (25.0)	11 (55.0)	3.808	0.149
marked improvement, n (%)	9 (36.0)	10 (50.0)	5 (25.0)	2.698	0.259
cured, n (%)	2 (8.0)	4 (20.0)	1 (5.0)		0.359
clinical efficacy, n (%)	11 (44.0)	14 (70.0)	6 (30.0)	6.636	0.036

Although no significant between-group differences were observed in overall improvement magnitudes (*P* > 0.05), the clinical efficacy rates (NAPSI 60 achievement) for nail lesions differed markedly: 44% (SEC), 70% (IXE), and 30% (GUS). Pairwise comparisons revealed that IXE demonstrated a significantly higher clinical efficacy rate compared to GUS (*P* = 0.011), while no significant differences were observed between SEC and GUS (*P* = 0.336) or SEC and IXE (*P* = 0.081) (**Figure 1F**).

### 3.2 Dermatoscopic characteristics of nail lesions

**Figure 2:**
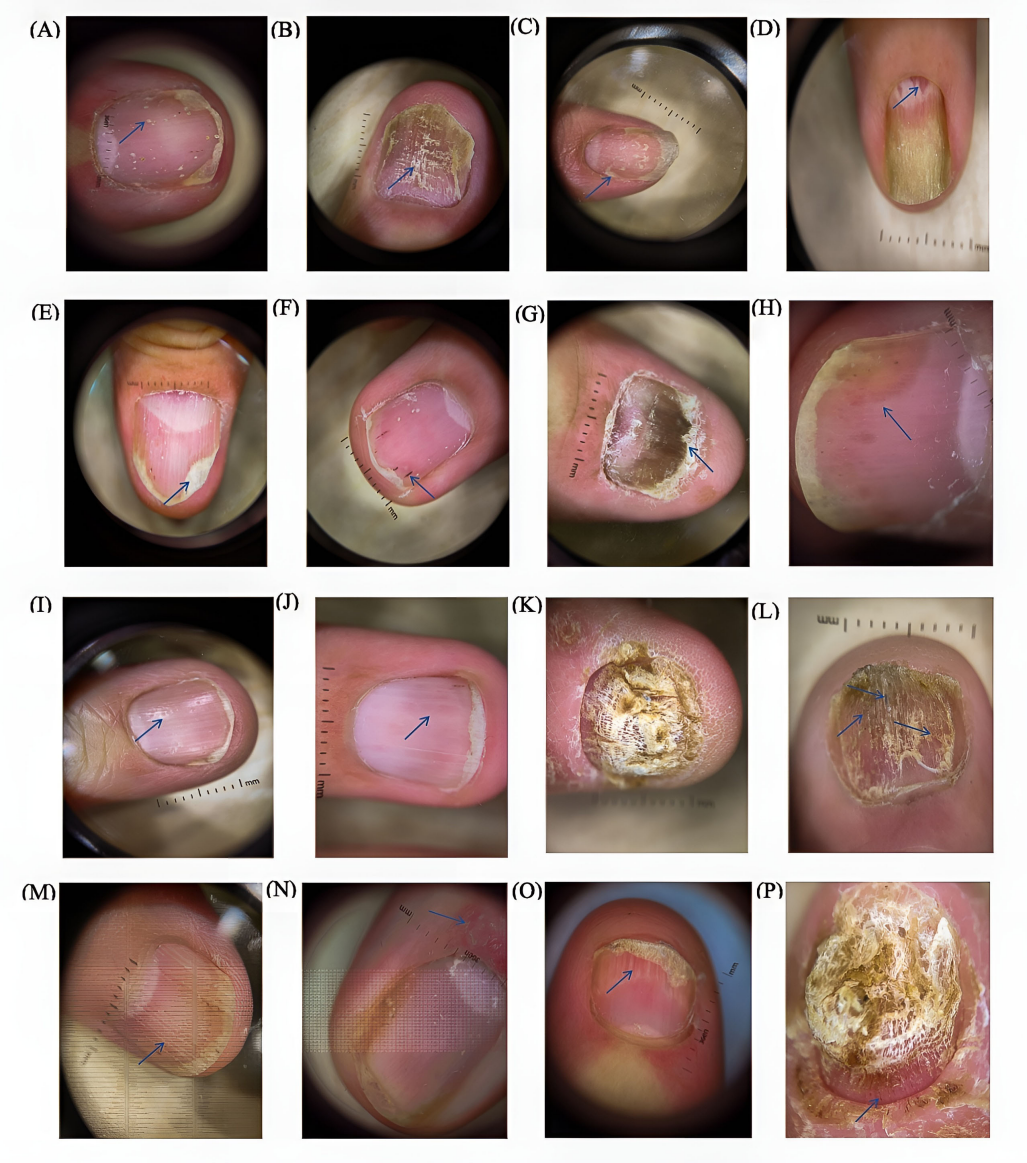
The four characteristics of nail matrix involvement: **(A)** pitting, **(B)** onychodystrophy, **(C)** leukonychia, **(D)** red spots in the lunula. The four characteristics of nail bed involvement: **(E)** onycholysis, **(F)** splinter hemorrhages, **(G)** subungual hyperkeratosis, **(H)** oil-drop discoloration. Two modes of dermoscopy: **(I)** non-polarized light, **(J)** polarized light. A patient with severe onychodystrophy: **(K)** before treatment, **(L)** after treatment. Four other features were observed: **(M)** longitudinal ridging, **(N)** proximal nail fold capillary dilation, **(O)** nail bed capillary dilation, **(P)** pseudo-fibrosis.

In this study, a total of 650 nail images from patients were collected using dermoscopy, with 458 nails showing lesions, accounting for 70.46% of all examined nails. The four characteristics of matrix involvement observed under dermoscopy are shown in **Figures 2A–D**: pitting in 249 nails (54.37%), onychodystrophy in 157 nails (34.28%), leukonychia in 68 nails (14.85%), and red spots in the lunula in 37 nails (8.08%). The four characteristics of bed involvement observed under dermoscopy are shown in **Figures 2E–H**: onycholysis in 231 nails (50.44%), splinter hemorrhages in 198 nails (43.23%), subungual hyperkeratosis in 126 nails (27.51%), and oil-drop discoloration in 98 nails (21.40%).

Under non-polarized light, finer pitting was more easily detected (**Figure 2I**), while under polarized light, finer oil-drop discolorations were more evident (**Figure 2J**).

In a nail with severe onychodystrophy (**Figure 2K**), the damage was present in all four quadrants. After treatment, the onychodystrophy significantly improved, revealing splinter hemorrhages (**Figure 2L**), but still occupying all four quadrants.

In addition to the eight features included in the NAPSI scoring, this study also observed four additional features (**Figures 2M–P**): longitudinal ridging in 39 nails (8.52%), proximal nail fold capillary dilation in 31 nails (6.77%), nail bed capillary dilation in 44 nails (9.61%), and pseudo-fibrosis in 13 nails (2.84%).

### 3.3 Efficacy of skin lesion treatment

#### 3.3.1 PASI

**Figure 3:**
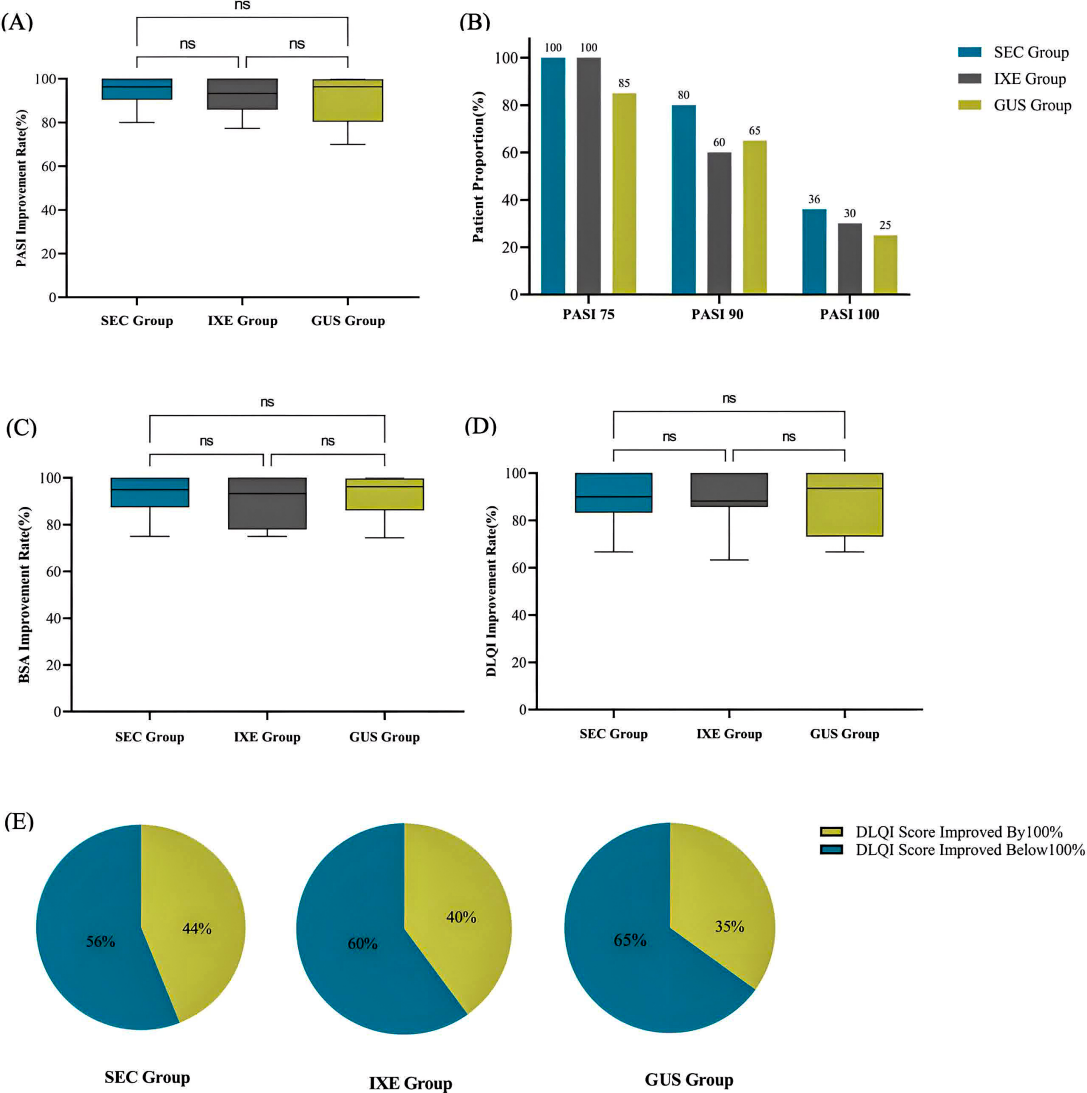
**(A)** PASI Improvement Rate. **(B)** Proportion of patients achieving PASI75/90/100. **(C)** BSA Improvement Rate. **(D)** DLQI Improvement Rate. **(E)** Proportion of patients with complete improvement in quality of life. ns, no statistical significance.

At week 24, the average improvement rates in PASI scores were 96.4 (90.5, 100.0)% in the SEC group, 93.7 (86.0, 100.0)% in the IXE group, and 96.4 (80.4, 99.8)% in the GUS group, with no statistically significant differences between the groups (*H* = 1.861, *P* = 0.394) (**Figure 3A**).

There were no significant differences in the degrees of improvement between the three groups (**Figure 3B**, P values were 0.052, 0.312, and 0.726, respectively).

#### 3.3.2 BSA

At week 24, the average improvement rates in BSA scores were 95.0 (87.5, 100.0)% in the SEC group, 93.3 (78.0, 100.0)% in the IXE group, and 96.2 (86.2, 99.7)% in the GUS group, with no statistically significant differences between the groups (*H* = 1.148, *P* = 0.563) (**Figure 3C**).

### 3.4 Improvement in quality of life

At week 24, the average improvement rates in DLQI scores were 90.0 (83.3, 100.0)% in the SEC group, 88.2 (85.7, 100.0)% in the IXE group, and 93.5 (69.7, 100.0)% in the GUS group, with no statistically significant differences between the groups (*H* = 0.934, *P* = 0.627) (**Figure 3D**).

At week 24, the number and proportion of patients achieving complete quality of life improvement (i.e., DLQI improvement rate of 100%) were 11 patients (44%) in the SEC group, 8 patients (40%) in the IXE group, and 7 patients (35%) in the GUS group, with no significant differences between the groups (*X* ^2^ = 0.375, *P* = 0.829) (**Figure 3E**).

### 3.5 Treatment-emergent adverse events (TEAEs)

During the treatment process, a total of 3 TEAEs (12%) were reported in the SEC group, comprising 2 cases (8%) of eczematous rash and 1 case (4%) of urticaria. In the IXE group, 4 TEAEs (20%) were documented, including 3 cases (15%) of injection site reactions and 1 case (5%) of urticaria. No TEAEs were observed in the GUS group.

## 4 Discussion

### 4.1 Analysis of the correlation between different dermatoscopic phenotyping of psoriatic nail lesions and the efficacy of biologics

In 2019, a consensus from a group of dermatologists and nail experts provides treatment recommendations for NP based on the severity of nail involvement and the specific area affected, whether the nail matrix or nail bed (4). Mild NP is defined as the involvement of ≤3 nails. For matrix-only involvement, intralesional corticosteroid injections are recommended as the first-line treatment. In cases of mild NP with involvement of the nail bed only, first-line treatments include intralesional corticosteroid injections, topical corticosteroids, topical vitamin D derivatives combined with corticosteroids, topical retinoids, and topical 0.1% tacrolimus. These therapies have demonstrated efficacy in treating nail bed disease. However, it is widely recognized that topical treatments may have limited effectiveness due to prolonged treatment duration, insufficient drug penetration through the nail plate, and challenges in maintaining patient compliance. This consensus offers valuable treatment guidance for NP patients with mild nail involvement in the absence of indications for systemic therapy. In the presence of psoriatic arthritis (PsA), systemic treatment should be considered, including options such as acitretin, methotrexate, cyclosporine, small molecule targeted drugs, and biologics. The Delphi consensus (20) recommends a dichotomous approach, categorizing patients into those requiring either topical or systemic treatment. Systemic treatment should be initiated if any of the following criteria are met: (1) body surface area (BSA) >10%; (2) involvement of special sites, such as the face, palms and soles, genital area, scalp, or nails; (3) failure of topical treatment. This model is of considerable significance in clinical practice and clinical trials for new therapies, reflecting a patient-centered approach to care.

In recent years, significant progress has been made in the use of biologics for the treatment of NP. By targeting specific molecules such as TNF-α, IL-17, IL-12/23, and IL-23, biologics have demonstrated substantial improvements in NP lesions. Among these treatment options, ixekizumab has shown the highest efficacy. A network meta-analysis (13) compared the complete cure rates of NP at 24-26 weeks among six approved biologics. The results indicated that ixekizumab had the highest cure rate at 46.5%. The cure rates for the other biologics were as follows: brodalumab (37.0%), adalimumab (28.3%), guselkumab (27.7%), ustekinumab (20.8%), and infliximab (0.8%). These findings align with another network meta-analysis (21) evaluating ten different drugs, which also demonstrated that at 24-26 weeks, ixekizumab exhibited the highest efficacy in achieving 100% improvement in NAPSI scores and led in NAPSI score reduction compared to other treatments. Similarly, the latest network meta-analysis in 2023 (22) demonstrated that ixekizumab had the highest rate of complete nail lesion clearance. In five head-to-head trials (16), patients treated with ixekizumab achieved higher rates of complete skin and nail clearance as early as week 12 compared to those treated with etanercept, guselkumab, ustekinumab, and adalimumab, with this superiority maintained through week 52. These findings underscore ixekizumab’s significant, rapid, and sustained efficacy in treating both skin and nail lesions.

Our real-world cohort demonstrates that IL-17A inhibitors achieve superior nail psoriasis efficacy over the IL-23 inhibitor at 24 weeks, aligning with their potent Th17-axis blockade. Notably, ixekizumab exhibited distinct advantages in nail bed pathology, while secukinumab excelled in matrix lesions. These findings extend prior evidence by integrating dermoscopic phenotyping to reveal site-specific therapeutic superiority—a novel dimension absent in existing literature. To our knowledge, this is the first study linking dermoscopic features to differential biologic responses, proposing a phenotype-driven selection framework: secukinumab for matrix-predominant and ixekizumab for bed-predominant involvement.

Despite these advances, limitations include a modest sample size and short-term follow-up, potentially limiting generalizability. Future multicenter studies with extended observation periods are needed to validate our dermoscopy-guided algorithm and assess long-term outcomes. Additionally, mechanistic investigations exploring IL-17A’s preferential targeting of nail matrix/bed keratinocytes could further refine personalized strategies.

### 4.2 Analysis of dermatoscopic characteristics of nail lesions

A recent study has demonstrated that dermoscopy is an effective, supportive, and non-invasive method that enhances the diagnosis of nail psoriasis (23). This study observed that the most common dermoscopic feature of nail psoriasis was pitting. In addition to NP, pitting can also occur in conditions such as alopecia areata and eczema, with distinct differences: pitting in alopecia areata is typically smaller, more regular in shape and distribution, while pitting in eczema tends to be coarser and more irregular (24). Dermoscopic examination is valuable in identifying these subtle differences, thereby aiding in the differential diagnosis.

In addition to the eight features included in the NAPSI (Nail Psoriasis Severity Index) score, this study identified four additional features: longitudinal ridging (8.52%), proximal nail fold capillary dilation (6.77%), nail bed capillary dilation (9.61%), and pseudo-fibers (2.84%). Chauhan (25) noted that longitudinal ridging is indicative of matrix involvement, with an incidence of 57.33% in fingernails and 22.77% in toenails, which is higher than the incidence observed in this study. The study also documented changes in the proximal and lateral nail folds, including scales, punctate capillary dilation, enlarged capillary dilation, and pustules. Long (6) not only observed these two features but also identified additional characteristics such as longitudinal fissures, transverse grooves, striped capillary dilation, and expanded capillary dots. International scholars (26) suggest that nail bed capillary dilation correlates with disease severity, with increased capillary density associated with more severe cases and reduction in capillary dilation observed following effective treatment. Yorulmaz (27) was the first to describe the phenomenon of pseudo-fibers, hypothesizing that these structures originate from the nail bed capillary network. These thread-like structures, found beneath the corneal layer, the distal free edge of the nail, or in areas where the nail plate has detached, resemble adherent fibers, which is why they are named pseudo-fibers. The color of these lesions corresponds to the arterial and venous ends of the capillaries, appearing red and black, respectively.

This study found that under non-polarized light, small nail pits were more easily observed, whereas under polarized light, subtle features such as oil-drop spots and splinter hemorrhages were more apparent. Consequently, non-polarized light is more effective for observing superficial nail plate lesions, such as pitting, crumbling, and subungual hyperkeratosis, while polarized light provides clearer visualization of deeper lesions with color changes, like splinter hemorrhages and oil-drop spots. Therefore, the combined use of both dermoscopic modes is recommended for a comprehensive assessment of nail pathology.

In this study, a nail exhibiting severe crumbling and scaling across all four quadrants had a pre-treatment NAPSI score of 4. Post-treatment, despite significant improvement in crumbling and scaling, the nail still affected all four quadrants, resulting in an unchanged NAPSI score of 4. This suggests that while the NAPSI score is a valuable tool for assessing the severity of nail psoriasis, it has limitations and may underestimate the actual treatment effects. Another assessment method, the Fingernails-Physician’s Global Assessment (F-PGA) (28), scores nail bed and matrix lesions on a scale from 0 to 4, with higher scores reflecting more severe nail involvement. However, the F-PGA method also has limitations; when there is a discrepancy between nail bed and matrix scores, the higher score is used as the F-PGA score, potentially overlooking other important lesion characteristics. In summary, while various scoring methods for nail psoriasis have their respective advantages and limitations, the NAPSI score remains an effective tool for assessing the severity of nail psoriasis lesions, encompassing both matrix and nail bed involvement. Its simplicity and comprehensiveness have led to its widespread use in both clinical and research settings. Additionally, our observations revealed that post-treatment improvements in nail crumbling exposed underlying features such as splinter hemorrhages, underscoring the value of dermoscopic assessment. Dermoscopy complements visual examination, providing a more objective and detailed evaluation of nail pathology.

### 4.3 Analysis of the correlation between psoriatic lesions and the efficacy of biologics

A retrospective cohort study in Korea (15) also demonstrated that at weeks 16 and 56, a higher proportion of patients treated with secukinumab achieved PASI 75 and PASI 90 compared to those treated with guselkumab and ustekinumab. However, at week 56, a higher proportion of guselkumab-treated patients achieved PASI 100, suggesting that while secukinumab offers superior early efficacy, guselkumab delivers more stable and effective long-term results. Similar conclusions were drawn by Reich et al. (18) Since this study includes only a 24-week follow-up, long-term efficacy data for guselkumab are not yet available. Further long-term observations are necessary to evaluate its sustained efficacy. Additionally, a network meta-analysis (29) comparing the efficacy of over 20 systemic therapies for plaque psoriasis between weeks 8 and 24 found that IL-17A inhibitor resulted in a higher proportion of patients achieving PASI 90 compared to all other interventions, consistent with the findings of this study.

### 4.4 Analysis of quality of life improvement in patients with psoriasis

Improvement in DLQI scores signifies a substantial enhancement in patients’ quality of life, making it a key factor in evaluating treatment efficacy (30). Research has consistently shown that DLQI scores significantly decrease in psoriasis patients undergoing biologic treatments (31–33). In this study, after 24 weeks of treatment, patients treated with secukinumab, ixekizumab, and guselkumab showed a significant reduction in DLQI scores compared to baseline, indicating substantial improvements across various dimensions of their quality of life. Other study (34) have found that psychological factors are the most significant contributors to the overall quality of life in patients with NP. Therefore, we believe that biologic treatments can alleviate the psychosocial burden associated with psoriasis, including anxiety and depression, thereby improving patients’ quality of life on a broader scale.

### 4.5 Safety analysis of biologics

Psoriasis is typically linked to Th1 and Th17 cell-mediated inflammation, characterized by key cytokines such as TNF-α, IFN-γ, IL-12, IL-17, and IL-23. In contrast, atopic dermatitis (AD) is primarily associated with Th2 cell-mediated inflammatory responses, involving cytokines such as IL-4, IL-13, IL-22, and IL-31 (35). These differences in immune pathways reflect distinct types of immune responses and the activation of different inflammatory mechanisms, which typically prevent their concurrent occurrence in the same patient (36). However, recent literature has reported the emergence of psoriasis-like lesions in AD patients and AD-like lesions in psoriasis patients following the use of certain targeted therapies. For instance, dupilumab (37) and the JAK inhibitor upadacitinib (38), used for treating AD, have been reported to induce psoriasis-like changes, while TNF-α inhibitors (35), IL-17 inhibitors (39–41), IL-23 inhibitors (42), and IL-12/23 inhibitors (43) have been associated with the development of AD-like lesions in patients undergoing treatment for psoriasis. This phenomenon is thought to be related to immune deviation (44), where the blockade of a specific pathway can cause a shift toward alternative immune pathways (40, 45), resulting in the coexistence of both diseases during treatment.

In this study, two cases of eczema-like rashes were observed in the SEC (Secukinumab) group during treatment, accounting for 8% of this treatment group, whereas no such adverse reactions were noted in the other two biologic groups. Further studies with larger sample sizes and extended follow-up periods are necessary to evaluate the differences in immune deviation among various biologics.

Research indicates that the citrate buffer and osmolarity adjusters, such as sodium chloride, are primary contributors to the injection site pain and swelling associated with ixekizumab (46, 47). The mildly acidic nature of the active drug, combined with the subcutaneous injection method, also stimulates superficial skin receptors, contributing to the pain experienced during administration. To address these issues, ixekizumab has upgraded its formulation by removing the citrate buffer and replacing the osmolarity adjuster with 80 mg/mL sucrose. This modification has significantly reduced local injection site reactions, thereby enhancing patient satisfaction and compliance (46, 48).

## 5 Conclusion

In this real-world prospective cohort study, IL-17A inhibitors (SEC and IXE) and the IL-23 inhibitor GUS demonstrated significant efficacy in improving both nail and skin lesions in patients with plaque psoriasis. Notably, IL-17A inhibitors exhibited superior overall efficacy compared to GUS, with distinct site-specific advantages: SEC showed greater improvement in dermoscopic nail matrix changes, while IXE was more effective for nail bed pathology. All treatments significantly enhanced patients’ quality of life and maintained favorable safety profiles, with no serious adverse events reported. These findings suggest that dermoscopic phenotyping may guide personalized biologic selection—SEC for matrix-predominant and IXE for bed-predominant nail psoriasis—while GUS remains a viable option for patients prioritizing safety. Further studies are warranted to validate these observations in larger, multicenter cohorts and explore long-term outcomes.

## Data Availability

The original contributions presented in the study are included in the article/**Supplementary Material**. Further inquiries can be directed to the corresponding author.
