# Supplementary material for: Targeting nail psoriasis: IL-17A inhibitors demonstrate site-specific superiority over IL-23 inhibitor in a 24-week dermoscopy-guided real-world cohort
Source: Front Immunol. 2025 Apr 8;16:1573715. doi: 10.3389/fimmu.2025.1573715 (PMC12011727; doi:10.3389/fimmu.2025.1573715)
Supplement: Supplementary file 1 [file Table1.docx]

Supplementary Material

# Supplementary Table 1. The basic information of the three groups

**Table S1. The basic information of the three groups**

| basic information | SEC group (n=25) | IXE group(n=20) | GUS group(n=20) | *F*/*H*/*X^2^* value | *P* value |
| --- | --- | --- | --- | --- | --- |
| gender |  |  |  | 0.498 | 0.779 |
| male, n(%) | 17(68.0) | 13(65.0) | 15(75.0) |  |  |
| female, n(%) | 8(32.0) | 7(35.0) | 5(25.0) |  |  |
| age (years), mean ± SD | 42.9 ± 12.5 | 40.0 ± 8.9 | 49.2(±16.0) | 1.847 | 0.168 |
| BMI (kg/m^2^), mean ± SD | 23.6(±3.6) | 24.5(±2.7) | 24.8 ± 3.4 | 0.439 | 0.647 |
| course (years), median(P25, P75) | 10.0(3.0,21.0) | 15.0(11.0,19.0) | 13.0(7.0,20.5) | 3.694 | 0.158 |
| PASI score, median(P25, P75) | 11.2(5.0,15.3) | 10.1(4.4,21.7) | 11.8(4.5,16.6) | 0.165 | 0.921 |
| BSA (%), median(P25, P75) | 10.0(6.5,19.0) | 11.0(6.0,28.7) | 13.0(7.3,20.2) | 0.398 | 0.820 |
| DLQI score, median(P25, P75) | 10.0(8.0,17.5) | 12.5(8.0,23.3) | 14.0(8.0,22.5) | 0.994 | 0.608 |
| pitting, median(P25, P75) | 19.0(8.5,38.5) | 22.0(15.0,25.0) | 28.0(12.0,40.0) | 0.847 | 0.655 |
| onychodystrophy, median(P25, P75) | 14.0(7.0,36.0) | 15.0(3.0,25.0) | 11.0(4.0,31.0) | 0.143 | 0.931 |
| leukonychia, median(P25, P75) | 9.5(5.0,22.0) | 5.0(2.5,13.0) | 6.5(3.0,16.0) | 0.976 | 0.614 |
| red spots in the lunula, median(P25, P75) | 10.0(2.5,18.5) | 8.0(1.0,16.0) | 11.0(4.0,17.0) | 0.632 | 0.729 |
| nail matrix NAPSI, median(P25, P75) | 47(15.5,74.0) | 35.0(22.5,43.5) | 36.5(17.3,59.8) | 0.150 | 0.712 |
| onycholysis, median(P25, P75) | 17.0(11.0,20.0) | 18.0(12.0,20.0) | 19.0(13.5,26.0) | 1.046 | 0.593 |
| splinter hemorrhages, median(P25, P75) | 15.0(1.0,19.0) | 11.0(5.0,18.0) | 13.0(1.5,16.0) | 1.456 | 0.483 |
| subungual hyperkeratosis, median(P25, P75) | 12.0(1.0,20,0) | 14.0(2.0,18.0) | 14.0(4.5,21.5） | 1.398 | 0.497 |
| oil-drop discoloration, median(P25, P75) | 13.0(3.0,7.5) | 11.0(2.0,12.0) | 9.5(3.5,17.0) | 3.153 | 0.207 |
| nail bed NAPSI, mean ± SD | 42.2 ± 26.4 | 49.7 ± 30.8 | 57.5 ± 36.2 | 1.084 | 0.346 |
| total NAPSI, mean ± SD | 85.1 ± 27.9 | 84.3 ± 32.4 | 91.0 ± 42.2 | 0.130 | 0.878 |

**
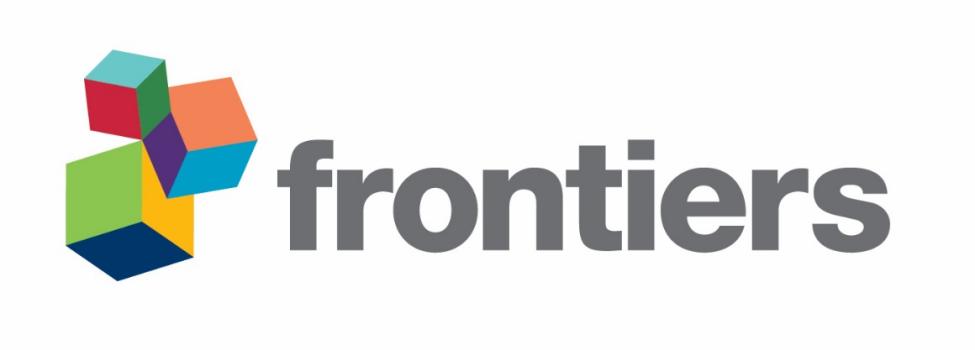
**
